# Identification of a multiple DAMP scavenger mimicking the DAMP-binding site of TLR4 to ameliorate lethal sepsis

**DOI:** 10.3389/fimmu.2025.1540908

**Published:** 2025-05-12

**Authors:** Takuya Murao, Gaifeng Ma, Atsushi Murao, Alok Jha, Jingsong Li, Yongchan Lee, Mian Zhou, Ping Wang, Monowar Aziz

**Affiliations:** ^1^ Center for Immunology and Inflammation, The Feinstein Institutes for Medical Research, Manhasset, NY, United States; ^2^ Departments of Surgery and Molecular Medicine, Zucker School of Medicine at Hofstra/Northwell, Manhasset, NY, United States

**Keywords:** DAMPs, eCIRP, HMGB1, H3, macrophage, phagocytosis, sepsis

## Abstract

Sepsis is a life-threatening organ dysfunction caused by a dysregulated host response to infection. Current treatments are limited to source control and supportive care, underscoring the urgent need for novel therapeutic interventions. Endogenous molecules released from stressed or damaged cells, known as damage-associated molecular patterns (DAMPs), exacerbate inflammation, organ injury, and mortality in sepsis. In this study, we discovered a novel therapeutic compound, opsonic peptide 18 (OP18), designed to scavenge multiple DAMPs, including extracellular cold-inducible RNA-binding protein (eCIRP), high mobility group box 1 (HMGB1) and histone H3, by facilitating their clearance via macrophages. OP18 was developed by identifying a 15-amino acid (aa) binding site within the extracellular domain of Toll-like receptor 4 (TLR4) shared by eCIRP, HMGB1, and histone H3, then extending it with an α_v_β_3_-integrin binding RGD (Arg-Gly-Asp) motif, resulting in an 18-aa peptide. Our data show that OP18 binds strongly to the above DAMPs and interacts with α_v_β_3_-integrin on macrophages, promoting phagocytosis of DAMPs and facilitating their lysosomal degradation. *In vitro*, OP18 reduced the production of the inflammatory cytokine TNF-α in DAMP-activated macrophages and restored mitochondrial function, as evidenced by improved oxygen consumption rate (OCR) and ATP production. In a lethal sepsis model induced by cecal ligation and puncture (CLP), DAMP levels were significantly elevated, while OP18 treatment markedly reduced the serum DAMP levels. Additionally, OP18-treated septic mice demonstrated reduced blood organ injury markers, decreased proinflammatory cytokine levels, attenuated ALI, and improved survival. These findings establish OP18 as a promising therapeutic molecule that reduces DAMP-induced inflammation, offering a potential strategy to improve outcomes in lethal sepsis.

## Introduction

Sepsis is defined as life-threatening organ dysfunction resulting from a dysregulated host response to infection (1). An estimated 50 million cases of sepsis and 10 million sepsis-related deaths occur worldwide annually, accounting for about 20% of all global deaths (2). Additionally, sepsis poses a substantial economic burden on US healthcare systems due to its high prevalence and need for intensive management, with annual costs exceeding $24 billion (3, 4). Despite these challenges, treatment options for sepsis remain limited to source control, antibiotics, and supportive care, highlighting the urgent need for novel therapies to improve patient outcomes.

Damage-associated molecular patterns (DAMPs) are endogenous molecules released from stressed or inflamed cells that induce aberrant immune responses, leading to organ injury via Toll-like receptor 4 (TLR4) in sepsis. Notable DAMPs include extracellular cold-inducible RNA-binding protein (eCIRP), high mobility group box 1 (HMGB1), and histones (5). CIRP is an 18-kDa RNA chaperone protein involved in regulating various cellular stress responses. When released extracellularly under inflammatory conditions, eCIRP acts as a DAMP, perpetuating inflammation and organ injury by activating TLR4 (6). Elevated circulating eCIRP levels are observed in conditions such as sepsis and hemorrhagic shock (6, 7). The binding of eCIRP to TLR4 triggers downstream signaling pathways that stimulate cytokine and chemokine production. HMGB1 is a nuclear protein composed of 215 amino acids capable of binding chromosomal DNA intracellularly to facilitate its nuclear functions, such as stabilizing nucleosomal structure and regulating gene expression (8, 9). Extracellular HMGB1 binds to TLR4 and receptors for advanced glycation end-products (RAGE) to exacerbate inflammation in sepsis (10). Histones, which are components of chromatin in the nucleus, can also act as DAMPs by binding to TLR4 when released into the extracellular space (5, 11). Histones are critical in the development of sepsis (12). Histone H3 citrullination promotes the formation of neutrophil extracellular traps (NETs) (13), which cause tissue injury. Citrullinated histone H3 is associated with relevant clinical outcomes in sepsis-acute respiratory distress syndrome (ARDS) patients and murine models of lung injury (14). Therefore, targeting DAMPs could be a novel therapeutic strategy for preventing inflammation in sepsis.

TLRs are pattern-recognizing receptors (PRRs) composed of a single transmembrane α-helix, which links the large ligand-binding domain at the N-terminus (60–90 kDa) to a cytoplasmic Toll–interleukin-1 receptor homology domain at the C-terminus (20 kDa) (15). Among the TLR family, TLR4 is one of the most extensively studied receptors, primarily expressed in immune cells of myeloid origin, including monocytes, macrophages, and dendritic cells (16). TLR4 initiates downstream signal transduction via myeloid differentiation primary response gene (MyD88) and Toll/interleukin-1 receptor (TIR) domain-containing adaptor protein (TIRAP) (17). This leads to mitogen-activated protein kinases (MAPKs) and nuclear factor-κB (NF-κB) activation, which subsequently promotes the release of inflammatory molecules. Dysregulated TLR4 signaling is a hallmark of immune disorders such as sepsis (18).

As a critical PRR in sepsis, TLR4 recognizes several DAMPs, such as eCIRP, HMGB1, and histones (19). This study aimed to develop a novel DAMP-scavenging strategy based on the shared binding site for multiple DAMPs on the extracellular domain of TLR4. We designed a small peptide derived from this TLR4 region to capture DAMPs. This peptide was then conjugated to a molecule recognized by phagocytic scavenging receptors, promoting efficient clearance of the resulting DAMP–peptide complex by phagocytes. Thus, targeting multiple DAMPs with a single molecular antagonist opens new therapeutic avenues for inflammatory diseases.

## Material and methods

### Animals

Male 8–12-week-old wild-type (WT) C57BL/6 mice were purchased from Charles River Laboratory (Charles River, Wilmington, MA, USA). Mice were housed in a temperature-controlled room on 12-h light cycles and provided standard laboratory chow and water. All experiments were performed following the guidelines for using experimental animals by the National Institutes of Health and were approved by the Institutional Animal Care and Use Committees (IACUC) of The Feinstein Institutes for Medical Research.

### Computational modeling and synthesis of OP18

The amino acid sequences of mouse TLR4 (Q9QUK6), mouse CIRP (P60824), mouse HMGB1 (P63158), and mouse histone H3 (P02301) were retrieved from the UniProt database. The structure models of CIRP, HMGB1, histone H3, and TLR4 were generated using a template-based modeling approach, Iterative Threading Assembly Refinement (I-TASSER) (20). The model structures were built using templates with maximum percentage identity, sequence coverage, and confidence. The models were refined based on repetitive relaxations by short molecular dynamics simulations for mild (0.6 ps) and aggressive (0.8 ps) relaxations with a 4-fs time step after structure perturbations. The model refinement enhanced certain parameters, including Rama-favored residues and a decrease in poor rotamers. The structure models of TLR4, HMGB1, CIRP, and histone H3 were aligned to get the structural similarity between all these proteins, and the structurally similar region was identified to design the peptide from the amino acid sequence of TLR4 using the PDBeFold tool. The peptide OP18 was docked into the structures of HMGB1, CIRP, and histone H3 using the GalaxyPepDock tool (21). The interactions between the complexes of HMGB1, CIRP, and histone H3 with OP18 were calculated using the PDBePISA tool. The proteins and protein-peptide complexes were visualized using PyMol and Chimera tools. OP18 was synthesized by GenScript (Piscataway, NJ, USA). A scrambled control peptide for OP18 (NRKINMGSFTQCSNNLGD) was designed using the online peptide sequence scrambler (https://peptidenexus.com/article/sequence-scrambler) and synthesized by GenScript.

### Surface plasmon resonance

Surface plasmon resonance (SPR) (OpenSPR, Nicoya, ON, Canada) was used to determine OP18’s direct interaction with recombinant mouse (rm) CIRP (eCIRP), recombinant human (rh)HMGB1, rhhistone H3, and rhα_v_β_3_ integrin. OP18 was synthesized by GenScript. rmCIRP was prepared in-house (7). rhHMGB1 (Cat. No.: 1690-HMB-050) was purchased from R&D System (Minneapolis, MN, USA). rhhistone H3 (Cat. No.: ab198757) was purchased from Abcam (Waltham, MA, USA). rhα_v_β_3_ integrin (Cat. No.: IT3-H52E3) was purchased from ACROBiosystems (Newark, DE, USA). OP18 was diluted to 10 µg/mL with the 10-mM acetate buffer pH 5 and immobilized to channel 2 in running buffer HBST (10 mM HEPES, 150 mM NaCl, 0.005% P20, 5 mM EDTA, pH 7.4). eCIRP (31.3–2,000 nM), HMGB1 (10–450 nM), α_v_β_3_ integrin (10–100 nM), and histone H3 (22.5–270 nM) were injected as analytes in the running buffer. The binding reactions were carried out at a flow rate of 40 µL/min at 20°C. Channel 1 was used to evaluate nonspecific binding. We optimized the OP18 ligand conditioning with 10 mM acetate buffer at pH 4–6 and found pH 5 provided the largest response. We used running buffer HBST pH 7.4 as a negative control. The real-time interaction data were analyzed by TraceDrawer (Nicoya). Data were globally fitted for 1:1 binding (one-to-one model).

### Treatment of macrophages with DAMPs and OP18

Mouse macrophage cell line RAW264.7 cells were obtained from American Type Culture Collection (Manassas, VA, USA) and cultured in Dulbecco’s modified Eagle’s medium (Thermo Fisher Scientific, Waltham, MA, USA), supplemented with 10% fetal bovine serum (FBS), 100 U/mL penicillin, and 100 μg/mL streptomycin and 1% glutamine in a 37°C incubator under humidified conditions containing 5% CO_2_ (22). Cells were plated and treated with rmeCIRP (1.0 μg/mL), rhHMGB1 (2.5 μg/mL), and rhhistone H3 (0.1 μg/mL) with or without 0.4 μM of scrambled peptide or OP18. After 20 h, culture supernatants were collected to measure TNF-α levels by ELISA.

### Seahorse XF Mito Stress Test assay

Seahorse XF Pro Analyzer and Seahorse Wave Pro Software (both from Agilent Technologies, Santa Clara, CA) were used for the metabolic assay. Seahorse XF RPMI (Cat. No.: 103576-100, Agilent Technologies) without Phenol Red supplemented with 2 mM glutamine, 10 mM glucose, and 1 mM pyruvate, pH 7.4 was used as a medium. Optimal seeding cell density was determined by the Seahorse XF Real-Time ATP rate assay (Cat. No.: 103592-100, Agilent Technologies). 10,000 cells/well of RAW264.7 cells were seeded in the Seahorse microplate two days before the assay. One day before the assay, RAW264.7 cells were treated with 0.5 µg/mL rmeCIRP, 2.5 µg/mL rhHMGB1, and 0.1 µg/mL rhhistone H3 in the presence and absence of 0.4μM OP18. Mitochondrial function was measured by Seahorse XF Cell Mito Stress Test Kit (Cat. No.:103015-100; Agilent Technologies) according to the manufacturer’s instructions. The concentrations of the compounds are as follows: oligomycin: 1.5 μM; phenylhydrazone (FCCP): 1μM; rotenone/antimycin: 0.5 μM. Wave data were analyzed with the Seahorse XF Mito Stress Test Report Generator.

### Immunofluorescence imaging and assays

To visualize cellular uptake of DAMPs by macrophages, RAW264.7 cells were treated for 4 h at 37°C with 1.0 μg/mL of FITC-conjugated eCIRP, DyLight 650-conjugated HMGB1, and Dylight 405-conjugated histone H3 protein with or without 0.4 μM of OP18. rmeCIRP was labeled with FITC Conjugation Kit (Fast) - Lightning-Link^®^ (Cat. No.: ab188285). rhHMGB1 was labeled with DyLight^®^ 650 Conjugation Kit (Fast) - Lightning-Link^®^ (Cat. No.: ab201803). rhhistone H3 was labeled with DyLight^®^ 405 Conjugation Kit (Fast) - Lightning-Link^®^ (Cat. No.: ab201798). Hoechst33342 or SYTO deep red nuclear stain (Invitrogen; S34900) was used for nuclear staining, and Lysotracker was used for lysosome staining. The cells were fixed with 4% formaldehyde and mounted with a Prolong Gold antifade. Confocal microscope images were acquired with ZEISS LSM 900 confocal microscopy (Oberkochen, Germany). The images were analyzed with ImageJ Fiji (National Institute of Health). Briefly, the Voronoi partition was created using a nuclear image. Each partition was converted to the region of interest and overlaid onto each fluorescence image. The mean fluorescence intensity of each cell in the image was measured.

### Mouse model of lethal sepsis

Polymicrobial sepsis was induced in mice by cecal ligation and puncture (CLP) (7, 23). Mice were anesthetized with isoflurane, and a midline abdominal incision was performed. The cecum was ligated with a 4–0 silk suture 1 cm proximal from its distal extremity and puncture using a 22-G needle was made twice (through and through) for a 20-h study and once for a survival study. A control (scrambled peptide) or OP18, both with 80 nmol/kg body weight (BW), was injected intraperitoneally (*i.p.*) before the closure. Sham animals were subjected to a laparotomy without CLP. Following the surgery, 1 mL of saline was injected subcutaneously to avoid surgery-induced dehydration, and 0.05 mg/kg buprenorphine was injected subcutaneously as an analgesic; 20 h after the surgery, the blood and lungs were collected. In the 20-h model, antibiotics were not administered, whereas in the survival study, 0.5 mg/kg BW of Imipenem was injected subcutaneously. Mice were observed for ten days for survival studies.

### Assessment of DAMPs, cytokines, and organ injury markers

Blood was collected, centrifuged at 1,000 × g for 10 min, and plasma or serum was separated. Plasma levels of eCIRP, HMGB1, and histone H3 were analyzed by ELISA kits according to the manufacturer’s instruction (eCIRP from American Research Products, Inc., Waltham, MA, USA, HMGB1 from Invitrogen, Thermo Fisher Scientific, and histone H3 from LS Bio, Shirley, MA, USA). Serum levels of IL-6 and TNF-α were analyzed by ELISA kits (IL-6 and TNF-α both from BD Biosciences, Franklin Lakes, NJ, USA) according to the manufacturer’s instruction. Serum levels of aspartate aminotransferase (AST), lactate dehydrogenase (LDH), blood urea nitrogen (BUN), and creatinine (Cre) were measured by specific colorimetric enzymatic assays (Pointe Scientific, Canton, MI). Absorbance was measured on a Synergy Neo2 (Agilent Technologies) according to the manufacturer’s instructions.

### Real-time quantitative RT-PCR

Total RNA was extracted from homogenized lung tissues using TRIzol reagent (Invitrogen, Thermo Fisher Scientific). cDNA was synthesized using murine leukemia virus transcriptase (Biosystems, Thermo Fisher Scientific), and PCR was performed with forward and reverse primers and SYBR Green PCR master mix (Biosystems) using a StepOne-Plus real-time PCR machine (Biosystems). The sequences of primers are as follows: β-actin, (forward) CGTGAAAAGATGACCCAGATCA, (reverse) TGGTACGACCAGAGGCATACAG; IL-6, (forward) CCGGAGAGGAGACTTCACAG, (reverse) GGAAATTGGGGTAGGAAGGA; TNF-α, (forward) AGACCCTCACACTCAGATCATCTTC, (reverse) TTGCTACGACGTGGGCTACA; IL-1β, (forward) CAGGATGAGGACATGAGCACC, (reverse) CTCTGCAGACTCAAACTCCAC; KC, (forward) GCTGGGATTCACCTCAAGAA, (reverse) ACAGGTGCCATCAGAGCAGT; MIP2, (forward) CCAACCACCAGGCTACAGG, (reverse) GCGTCACACTCAAGCTCTG.

### Lung myeloperoxidase assay

A total of 50 to 100 mg of lung tissues was liquid nitrogen-based homogenized in KPO_4_ buffer containing 0.5% hexadecyltrimethylammonium bromide (Sigma-Aldrich, St. Louis, MO, USA) using a sonicator with the samples placed on ice. Samples were subjected to two freeze/thaw cycles, and the supernatants were collected after the centrifugation (12,000×*g* for 15 min). The supernatants were diluted in a reaction solution containing *O*-dianisidine dihydrochloride (Sigma-Aldrich) and H_2_O_2_ (Thermo Fisher Scientific) as the substrate. Absorbance was measured at 460 nm, and myeloperoxidase (MPO) activity was calculated as the change in absorbance per minute divided by a gram of tissue.

### Lung histology

Lung tissues were fixed with 10% formalin and embedded in paraffin. Paraffin-embedded lung tissue blocks were sectioned at 5-μm thickness and placed on glass slides. Lung tissue sections were stained with hematoxylin and eosin (H&E) and observed with light microscopy. Lung injury scores were assessed using a scoring system established by the American Thoracic Society (24). Briefly, scores were evaluated from 0 to 1 based on the presence of neutrophils in the alveolar and interstitial spaces, hyaline membranes, proteinaceous debris in the airspaces, and alveolar septal thickening.

### Terminal deoxynucleotidyl transferase assay

To evaluate the presence of apoptotic cells in lung tissue, 5-μm lung tissue sections were stained using a commercially available terminal deoxynucleotidyl transferase (TUNEL) assay kit (*In Situ* Death Detection Kit, Roche Diagnostics, Indianapolis, IN, USA). Paraffin-embedded tissue sections were dewaxed in xylene, digested with proteinase K, washed, and incubated with an enzyme solution containing terminal deoxynucleotidyl transferase enzyme and fluorescence-labeled nucleotides at 37°C, according to the manufacturer’s protocol. 4′, 6-Diamidino-2-phenylindole (DAPI, Vectashield Antifade Mounting Media, H-2000) was used as a nuclear counterstain. Slides were examined under a ZEISS LSM 900 confocal microscopy. The TUNEL-positive cells in lung tissue sections were counted using ImageJ, Fiji software.

### Statistical analysis

Data analysis was performed using GraphPad Prism graphing and statistical software (GraphPad Software, LLC, San Diego, CA, USA). The presented data in the figures are expressed as mean ± SEM. Data was compared using one-way analysis of variance (ANOVA) followed by Tukey’s multiple comparison test for multigroups. An unpaired two-tailed Student’s *t*-test was applied for two group comparisons. Survival rates were analyzed by the Kaplan–Meier estimator and compared using a log-rank test. A *p*-value of < 0.05 was considered statistically significant for comparisons between experimental groups.

## Results

### Discovery of OP18 as a multi-DAMP targeting molecule

**Figure 1:**
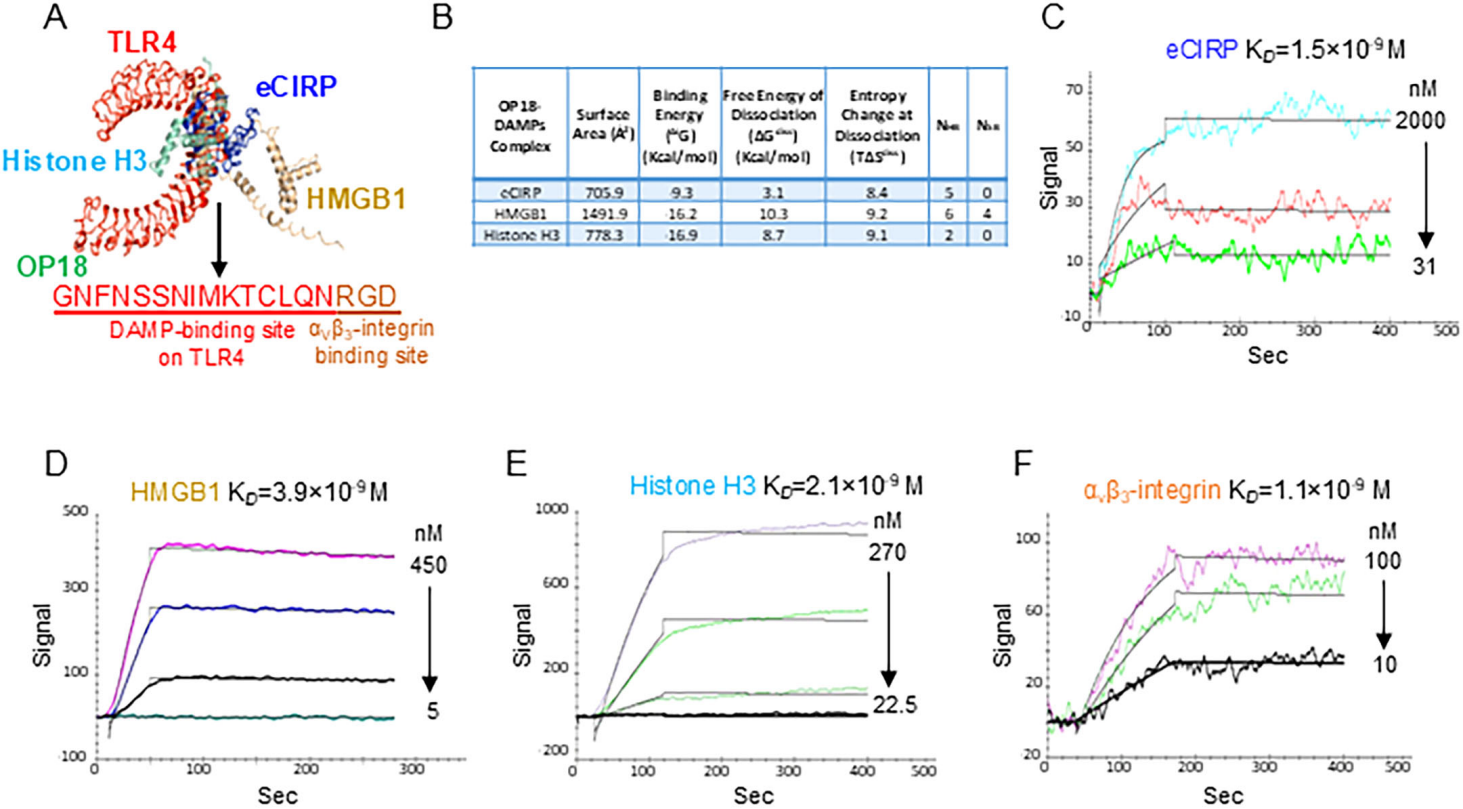
OP18 binds to multiple DAMPs with high affinity. **(A)** The common binding site of mouse TLR4 for mouse eCIRP, mouse HMGB1, and mouse histone H3 was determined by computational modeling, resulting in a 15 amino-acid (aa) sequence. Tagging an α_V_β_3_-integrin-binding site, RGD, to this 15-aa sequence resulted in an 18-aa peptide named OP18. **(B)** Computational prediction of molecular binding between OP18 and eCIRP, HMGB1, and histone H3. **(C–F)** BIAcore analysis of the binding capacity between OP18 and DAMPs or α_v_β_3_ integrin. BIAcore experiments were performed three times, and representative images and data are shown.

We first identified the common binding site on mouse TLR4 for mouse eCIRP, mouse HMGB1, and mouse histone H3 by computational modeling (**Supplementary Table S1**; **Supplementary Figure S1**). This 15-amino-acid sequence (GNFNSSNIMKTCLQN) was tagged with an Arginine–Glycine–Aspartate (RGD) motif to facilitate α_v_β_3_-integrin-dependent phagocytosis, resulting in an 18-amino-acid opsonic peptide (GNFNSSNIMKTCLQNRGD), named opsonic peptide 18 or OP18 (**Figure 1A**; **Supplementary Figure S2**). A 15-amino acid sequence within the leucine-rich repeat (LRR) region of TLR4 (**Supplementary Figure S3**) corresponds to OP18. Because OP18 does not bind to the TLR4 dimerization interface, it neither antagonizes TLR4 nor impairs downstream signaling crucial for host defense against infection (**Supplementary Figures S3**, **S4**). To investigate the binding potential between OP18 and eCIRP, HMGB1, and histone H3, we utilized a computational model to predict interactions between OP18 and these DAMPs. This revealed a strong interaction between OP18 and these DAMPs, as indicated by low binding energy (^Δi^G) and high free energy of dissociation (ΔG^diss^) (**Figure 1B**; **Supplementary Figure S5**). To confirm the binding between OP18 and these DAMPs and α_v_β_3_-integrin, we implemented surface plasmon resonance (SPR), also known as BIAcore assay. Consistent with the computational analysis, SPR revealed that OP18 bound to DAMPs with high affinity (**Figures 1C–E**). We also confirmed high binding affinity between OP18 and α_v_β_3_-integrin (**Figure 1F**). The amino acid sequence homology between mouse and human CIRP, HMGB1, and histone H3 is 95%, 99%, and 100%, respectively. In addition, the homology of the amino acid sequence of those DAMPs between mice and humans where OP18 recognizes is 100% (**Supplementary Figure S1**). This suggests that OP18 can equally recognize mouse and human eCIRP, HMGB1, and histone H3. Taken together, the novel peptide OP18 serves as a bridging molecule by recognizing multiple DAMPs and linking them to the phagocytic cells. We did not observe any cytotoxicity of OP18 on macrophages (**Supplementary Figures S6A, B**). OP18 did not induce immune responses as indicated by cytokine production in sham mice (**Supplementary Figures S6C, D**).

### OP18 scavenges multiple DAMPs by macrophages

**Figure 2:**
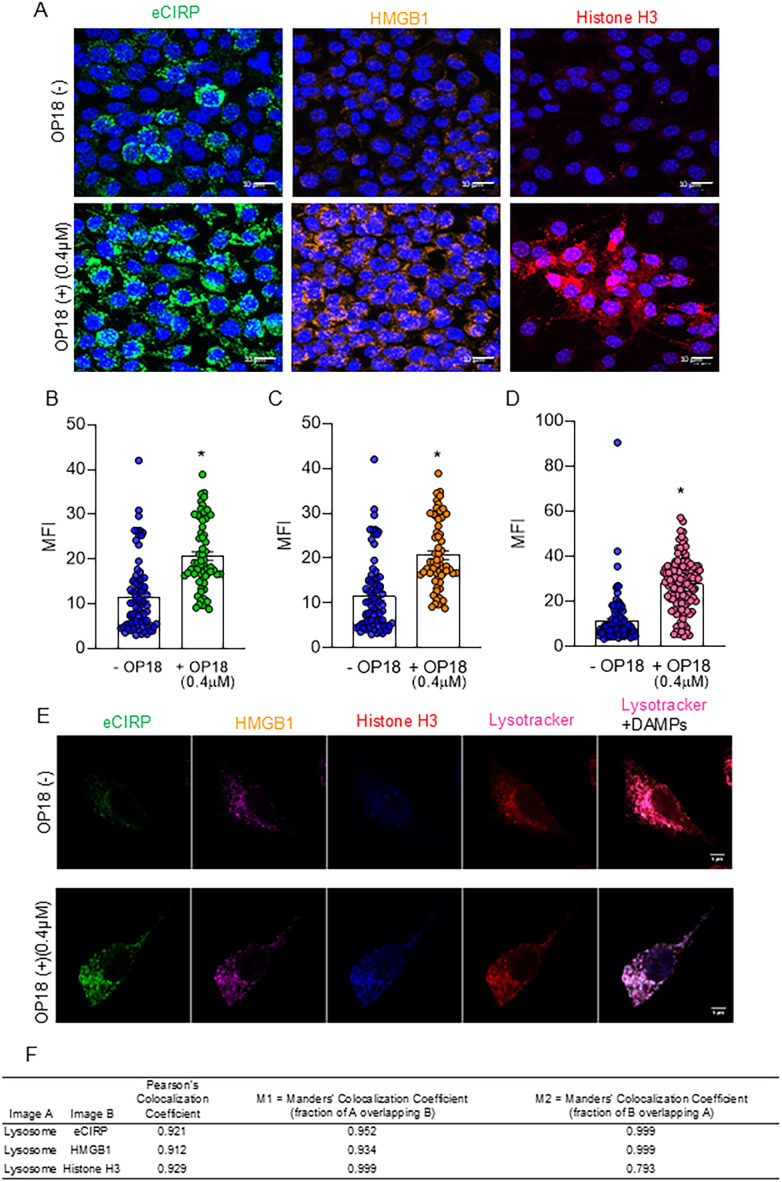
OP18 facilitates the clearance of multiple DAMPs by macrophages. RAW264.7 cells were incubated with 1.0 μg/mL of FITC-conjugated-rmeCIRP, Dylight 650-conjugated rhHMGB1, and Dylight 405-conjugated rhhistone H3 in the presence or absence of 0.4 μM OP18 for 4 h. **(A)** Representative confocal fluorescent microscopy images showing the fluorescence of labeled FITC-eCIRP (green), Dylight 650-HMGB1 (orange), Dylight 405-histone H3 (magenta), Hoechst33342 (blue), and SYTO Deep Red nucleic stain (blue). Quantitative analysis of confocal microscopy images. **(B–D)** Intracellular fluorescence intensity for each fluorochrome: **(B)** FITC-eCIRP, **(C)** Dylight 650-HMGB1, and **(D)** Dylight 405-histone H3. **(E)** Representative confocal fluorescent microscopy images of a single cell showing fluorescence of labeled FITC-eCIRP (green), Dylight 650-HMGB1 (magenta), histone H3 (blue), and Lysotracker (red). **(F)** Colocalization analysis of lysosomes and DAMPs using Pearson’s colocalization coefficient and Mander’s colocalization coefficient. Data are expressed as mean ± SEM and were compared using an unpaired two-tailed Student’s *t*-test. ^*^ *p* < 0.05 versus the group without OP18. Experiments were performed three times, and all data were included in the analysis.

To investigate whether OP18 promotes the internalization of eCIRP, HMGB1, and histone H3 from the extracellular space to the intracellular compartment and the degradation within the lysosomes in macrophages, we utilized macrophage-like RAW264.7 cells and fluorophore-labeled proteins, rmeCIRP-FITC, rhHMGB1-Dylight 650, and rhhistone H3-Dylight 405 to track DAMPs. Cells were also stained with Lysotracker to visualize lysosomes in the intracellular space. RAW264.7 treated with fluorophore-labeled DAMPs were incubated with or without OP18. After 4 h, a marked increase in DAMP uptake was visualized under confocal fluorescence microscopy in cells cultured with OP18 compared with cells cultured without OP18 (**Figures 2A–D**). These internalized DAMPs were observed within the lysosomes (**Figures 2E, F**). These results indicate that OP18 promotes the engulfment of DAMPs and subsequent degradation by lysosomes in macrophages.

### OP18 inhibits proinflammatory cytokine production and restores metabolic alteration in DAMP-treated macrophages

**Figure 3:**
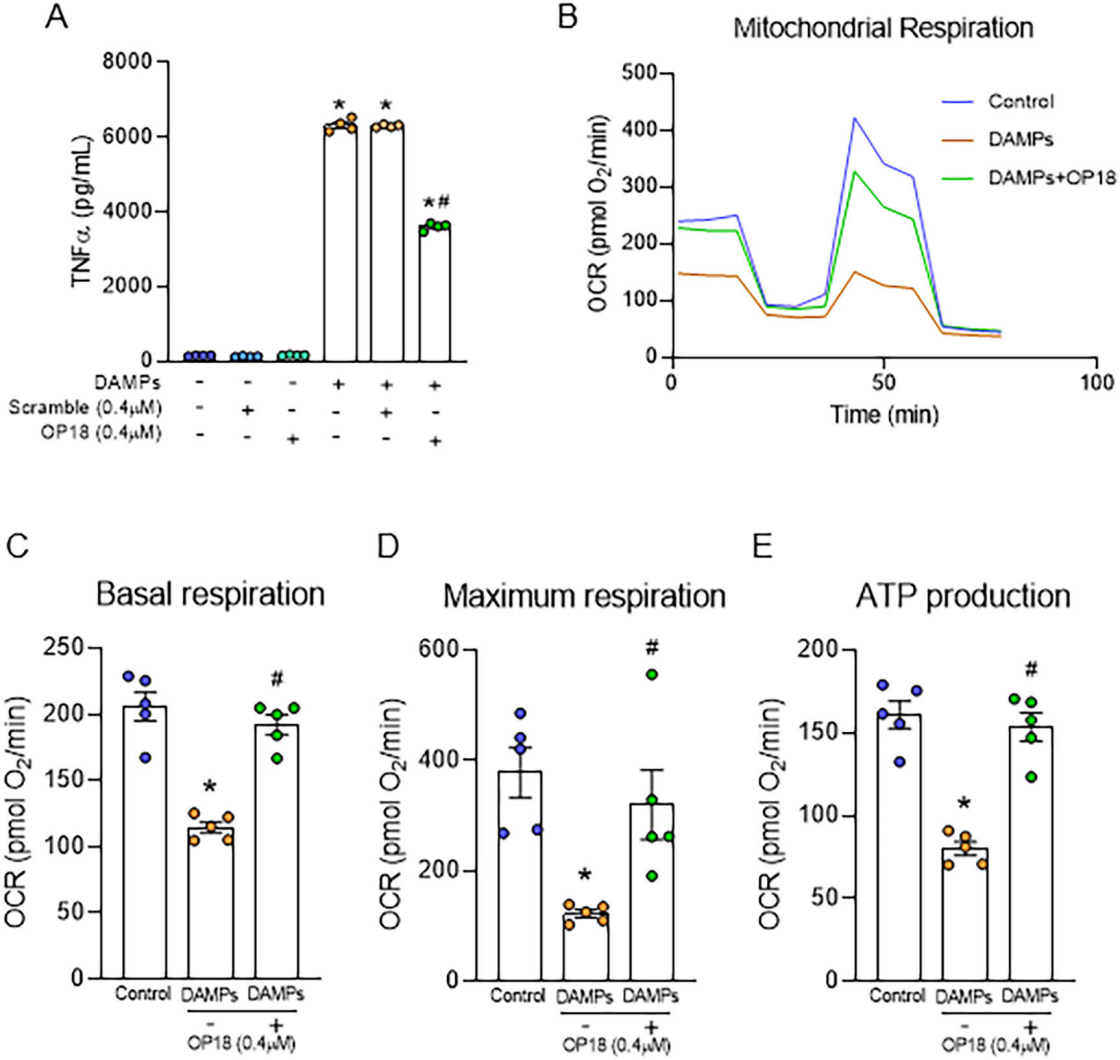
OP18 attenuates TNF-α production and corrects mitochondrial dysfunction in macrophages. **(A)** RAW264.7 cells were treated with DAMPs (1.0 μg/mL rmeCIRP, 2.5 μg/mL rhHMGB1, and 0.1 μg/mL rhhistone H3) in the presence of 0.4 μM OP18 or 0.4 μM scrambled peptide for 20 h, and supernatants were collected to assess TNF-α levels by ELISA. **(B–E)** RAW264.7 cells were treated with DAMPs (0.5 µg/mL rmeCIRP, 2.5 µg/mL rhHMGB1, and 1.0 µg/mL rhhistone H3) in the presence or absence of 0.4 μM OP18 overnight. Mitochondrial respiration and ATP production were measured. **(B)** Test profile; **(C)** maximum respiration; **(D)** basal respiration; **(E)** ATP production. Data are expressed as mean ± SEM (*n* = 4–5 samples/group) and were compared using one-way ANOVA followed by Tukey’s multiple comparison test. ^*^ *p* < 0.05 versus untreated control; ^#^ *p* < 0.05 versus DAMPs alone. Experiments were performed three times, and representative data are shown.

To investigate whether OP18 suppresses DAMP-induced proinflammatory response in macrophages, RAW264.7 cells were treated with rmeCIRP, rhHMGB1, and rhhistone H3 in the presence of OP18 or scrambled peptide. After 20 h, the levels of TNF-α in culture supernatants were determined. DAMPs treatment significantly increased the production of TNF-α from macrophages, whereas OP18 significantly reduced the production of TNF-α from macrophages treated by DAMPs compared to scrambled peptide stimulation (**Figure 3A**; **Supplementary Figures S7A–C**). Similarly, DAMP treatment significantly increased the production of IL-6 and TNF-α from THP-1 cells, whereas OP18 significantly reduced the production of both cytokines from THP-1 cells treated by DAMPs compared to scrambled peptide stimulation (**Supplementary Figures S7D, E**). Since mitochondrial metabolic alteration is associated with an inflammatory response in macrophages (25), we investigated the changes in mitochondrial metabolism in macrophages using Seahorse XF. Treatment of macrophages with DAMPs significantly reduced mitochondrial respiration in terms of basal and maximum respiration and ATP production, whereas OP18 significantly restored these metabolic parameters (**Figures 3B–E**). Taken together, OP18 suppresses proinflammatory cytokine production and restores metabolic homeostasis in macrophages.

### OP18 alleviates systemic inflammation and tissue injury in sepsis

**Figure 4:**
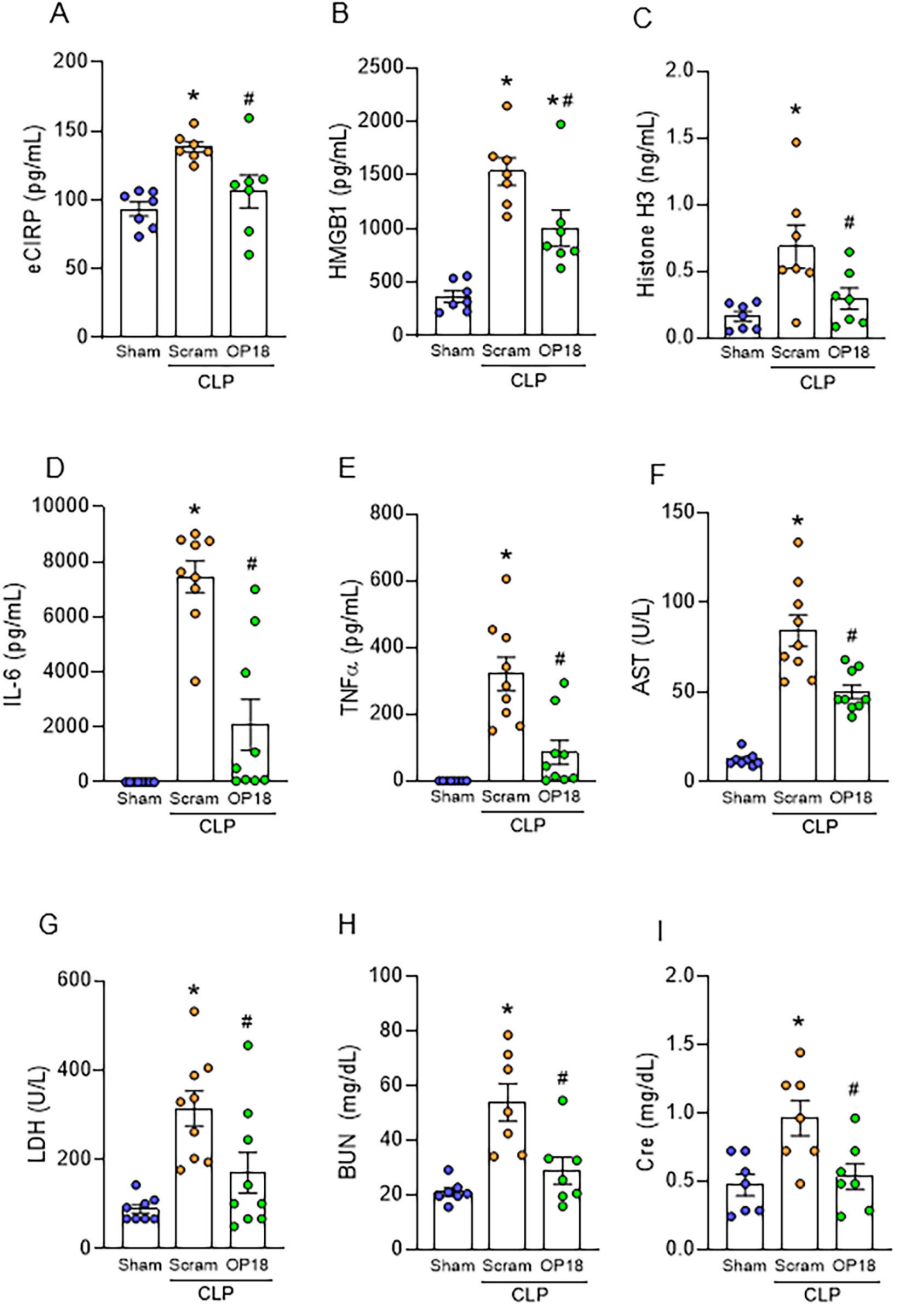
OP18 alleviates systemic inflammation and tissue injury in sepsis. Intraperitoneal injection of 80 nmol/kg scrambled peptide or 80 nmol/kg OP18 was performed on CLP mice before skin closure. Blood and lungs were collected 20 h after CLP. **(A–C)** Plasma levels of **(A)** eCIRP, **(B)** HMGB1, and **(C)** histone H3. **(D–I)** Serum levels of **(D)** IL-6, **(E)** TNF-α, **(F)** AST, **(G)** LDH, **(H)** BUN, and **(I)** Cre. Data are expressed as mean ± SEM (*n* = 7–9 samples/group) and were compared using one-way ANOVA followed by Tukey’s multiple comparison test. ^*^ *p* < 0.05 versus sham; ^#^ *p* < 0.05 versus scrambled peptide. Experiments were performed three times, and all data were used for analysis.

To evaluate the therapeutic effect of OP18 as a DAMP opsonic agent to improve outcomes in sepsis, we induced sepsis in WT mice using CLP and treated them with a one-time intraperitoneal injection of either a scrambled peptide or OP18 at the time of abdominal closure. Twenty hours after the surgery, the blood and lungs were harvested. We found that levels of DAMPs in the blood were significantly elevated after sepsis in mice treated with a scrambled peptide, whereas levels of DAMPs were significantly suppressed in OP18-treated mice (**Figures 4A–C**). We found that serum levels of IL-6, TNF-α, AST, LDH, BUN, and Cre were significantly elevated in septic mice injected with a scrambled peptide. In contrast, those parameters were dramatically suppressed in OP18-treated septic mice (**Figures 4D–I**). These results indicate that OP18 attenuates systemic inflammation and decreases organ injuries in sepsis.

### Treatment with OP18 attenuates acute lung injury and improves survival in sepsis

**Figure 5:**
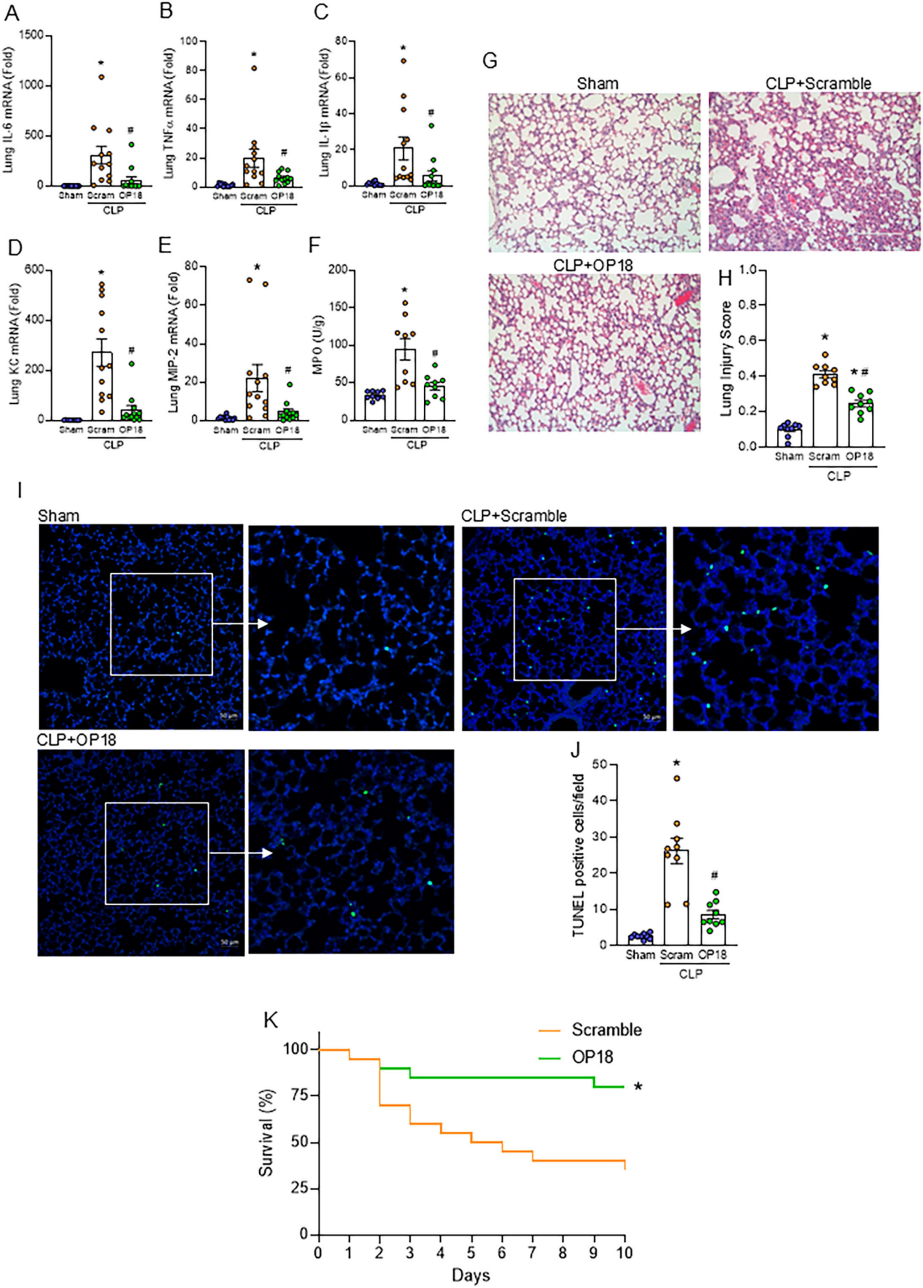
Treatment with OP18 attenuates acute lung injury and improves survival in sepsis. **(A–F)** mRNA levels of **(A)** IL-6, **(B)** TNF-α, **(C)** IL-1β, **(D)** KC, and **(E)** MIP-2, and **(F)** MPO activity in lungs. **(G)** Representative lung histology images of H&E staining; **(H)** lung injury score. **(I)** Representative images of TUNEL staining (green fluorescence) with nuclear counterstaining (blue fluorescence). **(J)** Numbers of TUNEL-positive cells/HPF in lung tissues. Original magnification, × 200; scale bars, 200 μm in H&E staining and 50 μm in TUNEL staining. Enlarged images of the boxed areas are shown to the right. Data are expressed as mean ± SEM (*n* = 9–12 samples/group) and were compared using one-way ANOVA followed by Tukey’s multiple comparison test. ^*^ *p* < 0.05 versus sham; ^#^ *p* < 0.05 versus scrambled peptide. Experiments were performed three times, and all data were used for analysis. **(K)** Ten-day survival study of septic mice induced by CLP with intraperitoneal injection of 80 nmol/kg of scrambled peptide or 80 nmol/kg of OP18 at the end of the procedure. *n* = 20 mice/group. Survival rates were analyzed by the Kaplan–Meier estimator using a log-rank test. ^*^ *p* < 0.05 versus scrambled peptide. Experiments were performed twice, and all data were included in the analysis.

**Figure 6:**
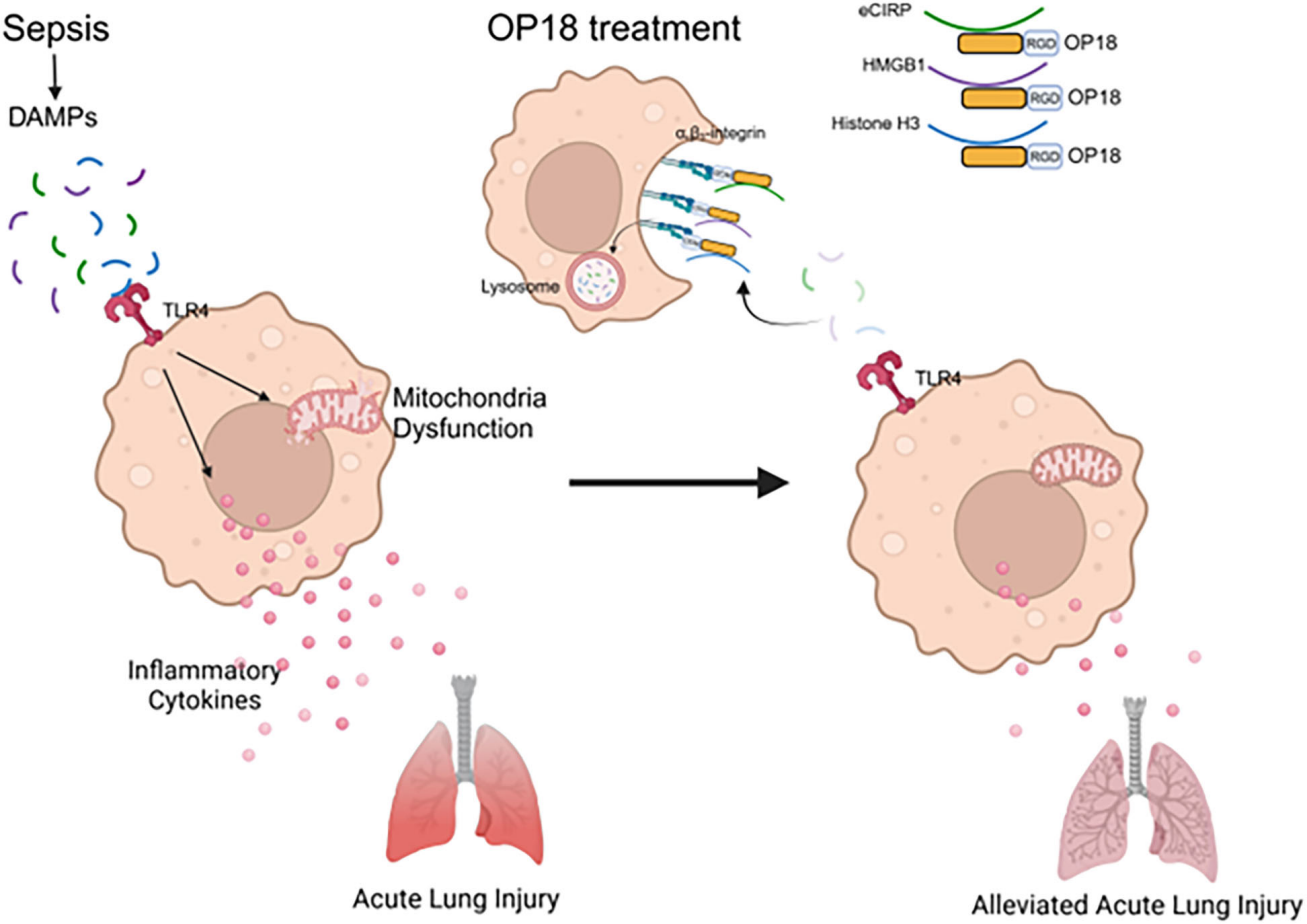
Summary. In sepsis, DAMPs stimulate TLR4, leading to increased production of inflammatory cytokines and metabolic reprogramming in mitochondria. OP18 harnesses DAMPs, promoting their clearance by macrophages and thereby attenuating systemic inflammation and ALI in sepsis.

We further evaluated the impact of OP18 on sepsis-induced ALI. Lung inflammation was induced by CLP as assessed by increased IL-6, TNF-α, IL-1β, keratinocyte chemoattractant (KC), and macrophage inflammatory protein-2 (MIP-2), as well as MPO activity in the lungs, whereas those parameters were significantly decreased in OP18-treated mice, indicating that OP18 mitigated lung inflammation and neutrophil influx in the septic lungs (**Figures 5A–F**). Next, we investigated the histological change of lungs in septic mice treated with a scrambled peptide or OP18. Histological analysis showed a severe tissue injury and an increase in apoptotic cells in the lung tissues of septic mice, whereas OP18 protected the lungs from those histological changes (**Figures 5G–J**). We then investigated the effect of OP18 on the survival of septic mice induced by CLP. Intraperitoneal OP18 administration at the end of surgery significantly improved survival in septic mice (**Figure 5K**). Together with the earlier findings, it is indicated that OP18 attenuates systemic inflammation by promoting the clearance of DAMPs and restoring mitochondrial function, alleviates ALI, and improves survival in sepsis (**Figure 6**).

## Discussion

Given the critical role of DAMPs in driving sepsis and the limited efficacy of targeting individual molecules/mediators to reduce sepsis-related mortality, we developed an opsonic drug, OP18, designed to simultaneously clear multiple DAMPs. In this study, we demonstrated that OP18 enhances the clearance of DAMPs by macrophages, suppressing the secretion of proinflammatory cytokines and alleviating mitochondrial dysfunction induced by DAMPs. Furthermore, our *in vivo* experiments revealed that administration of OP18 to septic mice significantly reduced the levels of circulating DAMPs attenuated systemic inflammation, organ injury, and ALI, and ultimately improved survival in sepsis.

TLR4 recognizes DAMPs and induces the activation and translocation of nuclear factor-kB, leading to the subsequent release of inflammatory cytokines such as TNF-α and IL-6 (26). A number of TLR4-targeting drugs have proven to have certain effects in animal models (27). However, an antagonist of TLR4-MD2 failed to demonstrate a significant effect on mortality in severe sepsis in clinical trials (28). This could potentially be due to the inhibition of TLR4-mediated immune response, which is indispensable for combating infection. An alternative approach to overcome this failure is to utilize therapeutics targeting DAMPs (ligands of TLR4). We have developed multiple drugs targeting eCIRP and have achieved some success in improving the mortality of sepsis. Particularly, in our previous study, we developed an opsonic drug that enables macrophages to clear eCIRP, successfully protecting septic mice from systemic inflammation, ALI, and mortality (22). However, it opsonizes only eCIRP, and other DAMPs may still affect the severity of sepsis. In addition, inhibition of HMGB1 or histones individually has not translated into clinical outcomes. Therefore, we aimed to develop a broad-spectrum opsonic agent capable of targeting multiple DAMPs simultaneously. Since eCIRP, HMGB1, and histone H3 are the major DAMPs that promote inflammation via TLR4 in sepsis, we selected them as targets to be cleared from circulation. OP18 was generated by using the shared binding site of TLR4 for these DAMPs and being tagged with an α_v_β_3_-integrin binding RGD motif. In addition to these DAMPs, since OP18 is derived from the TLR4 sequence, it could potentially bind other known or unknown TLR4-stimulating DAMPs, which would be the unique strength of this peptide.

DAMPs in general have multiple receptors in addition to TLR4, such as, but not limited to triggering receptors expressed on myeloid cells-1 (TREM-1), RAGE, and IL-6R (29). Thus, OP18 has an advantage over TLR4 antagonists and other DAMP receptor inhibitors in the sense that it could abrogate the activation of multiple receptors simultaneously by removing DAMPs. It has been shown that internalized DAMPs could facilitate the inflammatory pathways via cytosolic receptors. A recent study demonstrated that DAMPs like HMGB1 bound to LPS and caused LPS internalization via the RAGE receptor, activating caspase-11, a cytosolic LPS receptor (30). In this aspect, it is assumable that the HMGB1 containing LPS could be devoid of phagosome formation or not fusing to the lysosome for degradation. This would be the reason why the HMGB1-containing LPS could trigger intracellular LPS receptors to activate innate signaling. However, in our study, we confirmed that OP18 could capture DAMPs and internalize them, followed by merging them into lysosomes for degradation. This leads to the termination of any intracellular effects exerted by internalized DAMPs or accompanied LPS.

Studies have shown that in different stages of sepsis, especially in the early stage, the metabolic pathway of macrophages is significantly altered (31, 32). Activation of macrophages by pro-inflammatory stimuli switches glucose metabolism from oxidative phosphorylation (OXPHOS) to glycolysis (33, 34). Accumulation of metabolites produced by aberrant glycolysis induces the inflammatory response, leading to the secretion of IL-1β and TNF-α (35, 36). In our *in vitro* experiment, DAMPs reduced OXPHOS and ATP production in macrophages, accompanied by the increased production of TNF-α; however, OP18 restored OXPHOS and ATP reduction in macrophages and suppressed the production of TNF-α. The reduction of OXPHOS and ATP is considered to be the result of mitochondrial dysfunction (37, 38). DAMPs can increase the mitochondrial ROS generation and decrease mitochondrial membrane potential, leading to impairment of membrane integrity (39). OP18 prevents mitochondrial dysfunction induced by DAMPs, attenuating proinflammatory signaling mediated by damaged mitochondrial products.

In our model of sepsis, we did not explore different doses and treatment timing strategies. We administered OP18 once at the end of the surgical procedure. Additional dose and time course studies can be done in the future. It is usually not possible to treat patients with sepsis therapies at the time of the initial insult. However, it is challenging to diagnose sepsis accurately in mice without an invasive procedure, such as blood collection. Clinically, sepsis diagnosis is made by blood parameters and physiological conditions. Unfortunately, repeated blood collection is highly invasive in mice due to the lower amount of circulating blood. There remain limitations, such as variability in the development of sepsis, even if the same procedure was used in CLP. Moreover, CLP basically mimics intra-abdominal bacterial sepsis. It is critical to test the efficacy of OP18 in other sepsis models, such as pneumonia or viral infection. We did not address the pharmacokinetics of OP18 and its toxicity to the liver or kidney. We should also acknowledge that the experimental model of sepsis has limitations, and it may not represent the high complexity of the septic condition in humans. Thus, higher animal usage is necessary to confirm more clinically relevant therapeutic interventions. DAMPs are involved in sterile inflammation, such as hepatic ischemia/reperfusion or gut ischemia/reperfusion. OP18 can be potentially used for the treatment option of such sterile inflammation.

Levels of multiple DAMPs, i.e., eCRIP, HMGB1, and histone H3, were significantly decreased in septic mice by OP18 treatment, strongly indicating the efficacy of OP18 in DAMP clearance *in vivo*. Nevertheless, it is important to consider *in vivo* off-target effects, such as interaction with pathogens or molecules other than DAMPs. Computational modeling and BIAcore assays revealed a strong binding affinity between OP18 and DAMPs, with Kd values comparable to antigen–antibody interactions. This interaction is mediated by OP18’s recognition of a specific amino acid sequence on DAMPs. If pathogens or other molecules share this same amino acid sequence, the OP18 may also facilitate the clearance of these pathogens or molecules.

When developing anti-inflammatory drugs for sepsis, it is important not to interfere with anti-pathogen immunity. Our previous work demonstrated that eCIRP inhibits macrophage bacterial phagocytosis (40). Therefore, the observed reduction in DAMPs by OP18 is anticipated to enhance anti-pathogen immunity. DAMPs have also been shown to potentially facilitate tissue repair (41). While direct assessment of tissue repair was not performed in this study, the observed reductions in tissue injury and improvements in survival suggest that OP18 may confer a broader benefit in restoring organ function.

We set the culture time of the phagocytosis experiment for 4 h in this study based on a study published from our laboratory (22), where we evaluated the uptake of a DAMP eCIRP by an opsonic reagent via α_V_β_3_ integrin. Based on this work, we considered that the treatment of RAW264.7 cells with DAMPs and OP18 for 4 h can be optimal. OP18 is an 18-amino acid peptide (~ 3 kDa), so its molecular weight is much lower than that of eCIRP (18 kDa), HMGB1 (25 kDa), and histone H3 (15 kDa). Thus, the dose of OP18 used is theoretically sufficient to capture all DAMPs in the cell medium. Here, we only evaluated OP18-mediated DAMP clearance and cytokine inhibition by mouse macrophages *in vitro*. Moreover, these experiments were mostly performed in a cell line rather than in primary cells. It is important to confirm this opsonic and anti-inflammatory capacity of OP18 using primary cells as well as human macrophages for better clinical relevance.

We evaluated lung injury not only blindly by lung histology using a scoring system established by the American Thoracic Society but also by quantifying tissue cytokines and chemokines that reflect inflammation. It would be of interest to evaluate bronchoalveolar lavage fluid contents as well as conduct physiological tests, such as blood gas and oxygenation, compliance, and resistance measurement. We evaluated cell death in the lung tissues by TUNEL staining and observed a significant increase in TUNEL-positive cells by CLP and a decrease in OP18. Even though we did not specifically assess the cell type using cell markers, it has been shown that TUNEL-positive cells reflect cell death of epithelial cells and endothelial cells in the lungs of septic mice (42). The pathogenesis of sepsis-induced ALI highlights the role of epithelial and vascular cell death in contributing to organ injury (43). Sepsis, by definition, is a multi-organ dysfunction, making it difficult to narrow down the ultimate cause of death to one organ dysfunction. In addition to ALI, we observed elevated markers of liver and kidney injury in septic mice and amelioration of these markers with OP18 treatment. It would also be interesting to evaluate the efficacy of OP18 on other organs, such as the heart and central nervous system, both of which can be affected by sepsis.

In conclusion, OP18 promotes the clearance of multiple DAMPs simultaneously by macrophages, reducing the proinflammatory cytokines and chemokines in blood and lungs and improving survival rates in sepsis (**Figure 6**).

## Data Availability

The original contributions presented in the study are included in the article/**Supplementary Material**. Further inquiries can be directed to the corresponding author.
