# Supplementary material for: Identification of a multiple DAMP scavenger mimicking the DAMP-binding site of TLR4 to ameliorate lethal sepsis
Source: Front Immunol. 2025 May 12;16:1540908. doi: 10.3389/fimmu.2025.1540908 (PMC12104086; doi:10.3389/fimmu.2025.1540908)
Supplement: Supplementary file 1 [file DataSheet1.pdf]

Supplemental Table 1: Amino acid sequence of mouse DAMPs shared binding site on mouse TLR4

TLR4	eCIRP	HMGB1	Histone H3
Gly234	Val52	Pro118	His40
Asn235	Thr53	Gly119	Arg41
Phe236	Phe54	Leu120	Tyr42
Asn237	Glu55	Ser121	Arg43
Ser238	Asn56	Ile122	Pro44
Ser239	Ile57	Gly123	Gly45
Asn240	Asp58	Asp124	Thr46
Ile241	Asp59	Val125	Val47
Met242	Ala60	Ala126	Ala48
Lys243	Lys61	Lys127	Leu49
Thr244	Asp62	Lys128	Arg50
Cys245	Ala63	Leu129	Glu51
Leu246	Met64	Gly130	Ile52
Gln247	Met65	Glu131	Arg53
Asn248	Ala66	Met132	Arg54

Supplemental Figure 1: Sequence homology between mouse and human

CIRP mouse (upper; NP_001366350.1) and human (lower; NP_001271.1) amino acids sequence (168-aa) homology: 95%

```
Mouse: MASDEGKLFVGGLSFDTNEQALEQVFSKYGQISEVVVVKDRETQRSRGFGFVTFENIDDA
Human: MASDEGKLFVGGLSFDTNEQSLEQVFSKYGQISEVVVVKDRETQRSRGFGFVTFENIDDA

Mouse: KDAMMAMNGKSVDGRQIRVDQAGKSSDNRSRGYRGGSAGGRGFFRGGRSRGRGFSRGGGD
Human: KDAMMAMNGKSVDGRQIRVDQAGKSSDNRSRGYRGGSAGGRGFFRGGRGRGRGFSRGGGD

Mouse: RGYGGGRFESRSGGYGGSRDYYASRSQGGSYGYRSSGGSYRDSYDSYG
Human: RGYGGNRFESRSGGYGGSRDYYSSRSQSGGYSDRSSGGSYRDSYDSYA
```

HMGB1 mouse (upper; NP_001300823.1) and human (lower; NP_001300821.1) amino acids sequence (215aa) homology: 99%

```
Mouse: MGKGDPKKPRGKMSSYAFFVQTCREEHKKKHPDASVNFSEFSKKCSERWKTMSAKEKGKF
Human: MGKGDPKKPRGKMSSYAFFVQTCREEHKKKHPDASVNFSEFSKKCSERWKTMSAKEKGKF

Mouse: EDMAKADKARYEREMKTYIPPKGETKKKFKDPNAPKRPPSAFFLFCSEYRPKIKGEHPGL
Human: EDMAKADKARYEREMKTYIPPKGETKKKFKDPNAPKRPPSAFFLFCSEYRPKIKGEHPGL

Mouse: SIGDVAKKLGEMWNNTAADDKQPYEKKAAKLKEKYEKDIAAYRAKGKPDAAKKGVVKAEK
Human: SIGDVAKKLGEMWNNTAADDKQPYEKKAAKLKEKYEKDIAAYRAKGKPDAAKKGVVKAEK

Mouse: SKKKKEEEDDEEDEEDEEEEEEEEDEDEEEDDDDE
Human: SKKKKEEEEDEEDEEDEEEEEDEEDEDEEEDDDDE
```

Histone H3.1 mouse (upper; NP_038578.2) and human (lower; NP_003520.1) amino acids sequence (136aa) homology: 100%

```
Mouse: MARTKQTARKSTGGKAPRKQLATKAARKSAPATGGVKKPHRYRPGTVALREIRRYQKSTE
Human: MARTKQTARKSTGGKAPRKQLATKAARKSAPATGGVKKPHRYRPGTVALREIRRYQKSTE
```

```
Mouse: LLIRKLPFQRLVREIAQDFKTDLRFQSSAVMALQEACEAYLVGLFEDTNLCAIHAKRVTI
Human: LLIRKLPFQRLVREIAQDFKTDLRFQSSAVMALQEACEAYLVGLFEDTNLCAIHAKRVTI

Mouse: MPKDIQLARRIRGERA
Human: MPKDIQLARRIRGERA
```

Note: Amino acid sequence different between mouse and human is denoted in green color. Amino acid sequence of shared binding site on TLR4 is shown in red color.

Supplemental Figure 2: Binding between the 15-amino acid sequence on mouse TLR4 and each mouse DAMP

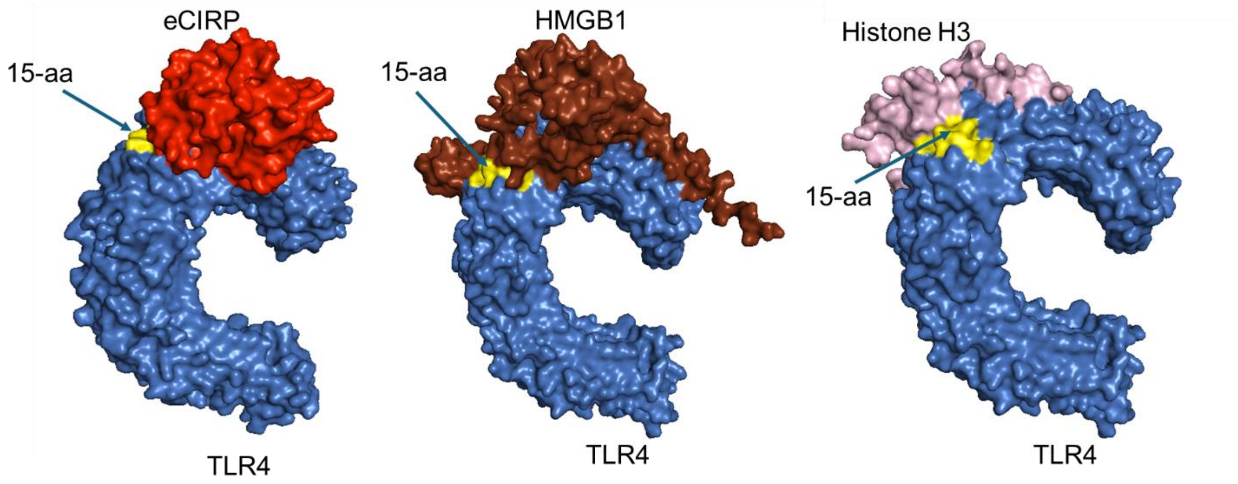

Supplemental Figure 3: Location of amino acid sequence of OP18's DAMP-binding site (aa 234-248) in TLR4 leucine-rich extracellular domain

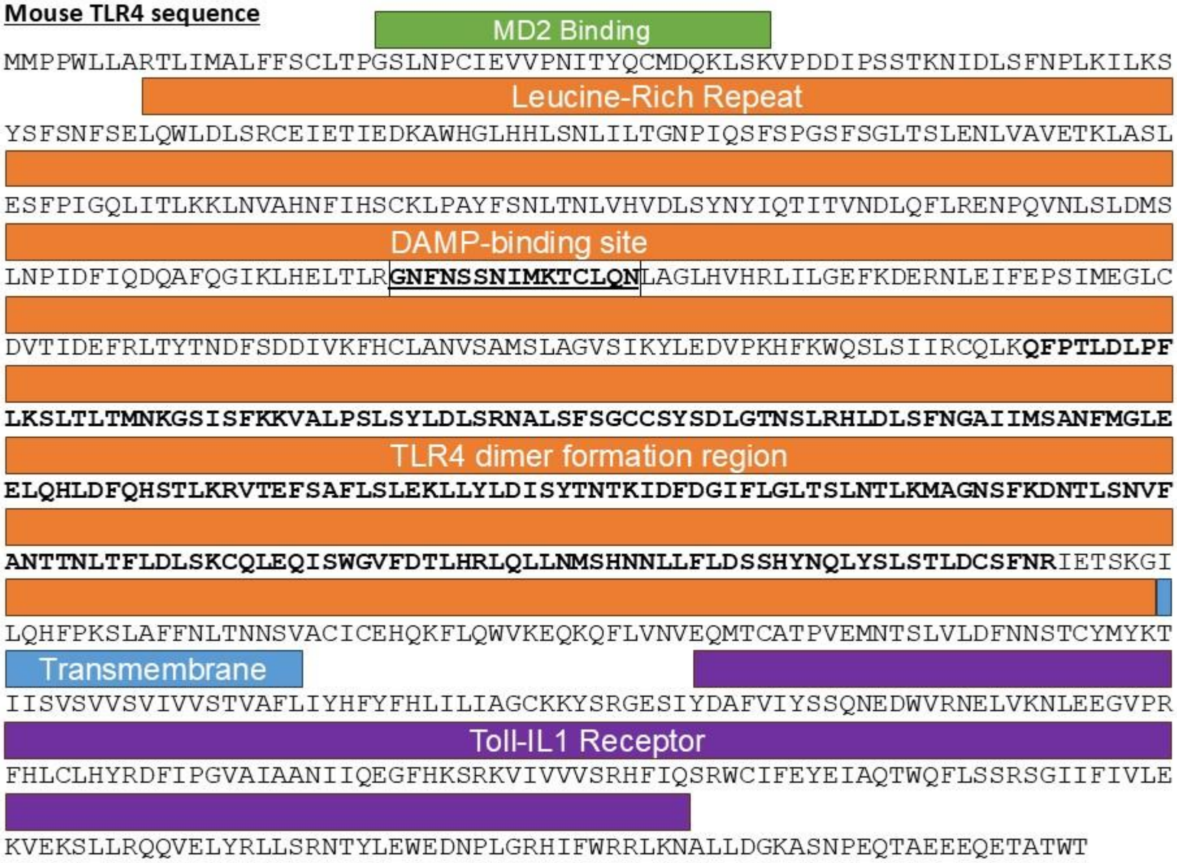

Supplemental Figure 4: mouse TLR4 dimer formation region (aa 342-553)

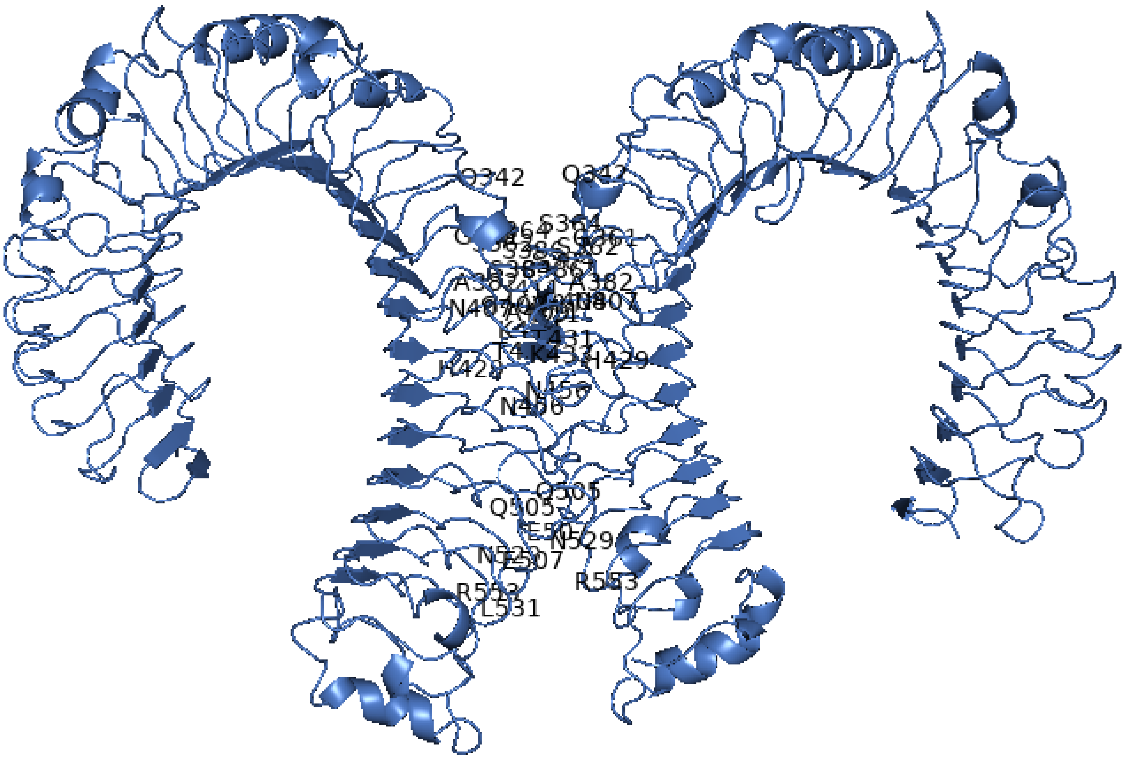

TLR4 Dimer Residues: Q342, G361, S362, S364, A382, S384, S386, N407, G408, A409, I411, H429, T431, K433, N456, Q505, E507, N529, L531, R553

Supplemental Figure 5: Binding between OP18 and each mouse DAMP

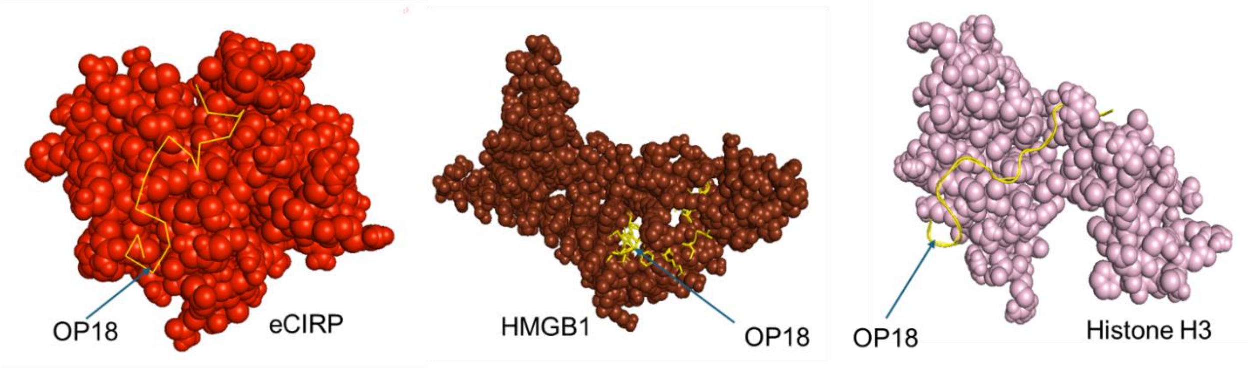

Supplemental Figure 6: Toxicity Assay of OP18

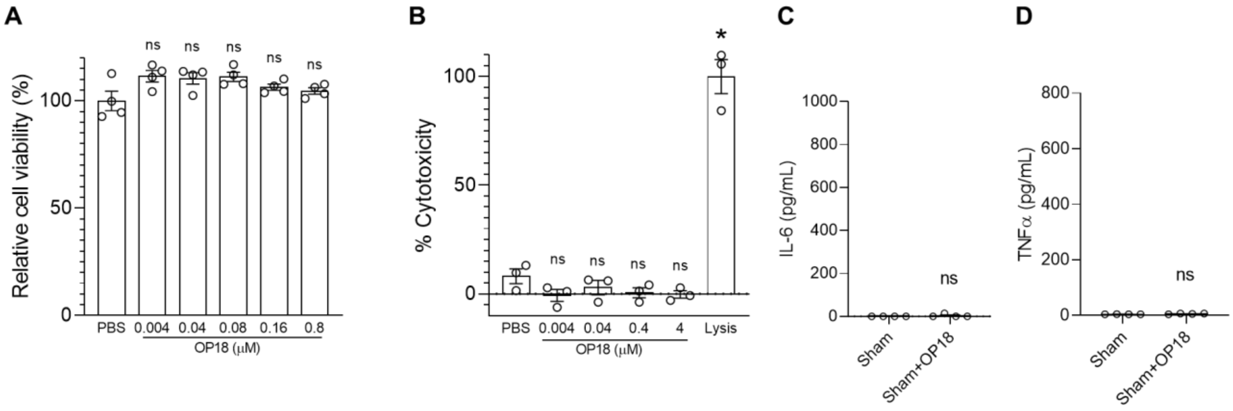

Note: (A) RAW264.7 cells were treated with OP18 for 20 h and MTS assay was performed using a CellTiter 96® AQ$_{ueous}$ Non-Radioactive Cell Proliferation Assay kit (Promega, Madison, Wisconsin) according to the manufacturer's instructions. **(B)** Mouse peritoneal macrophages were treated with OP18 for 20 h and CyQUANT™ LDH Cytotoxicity Assay (Thermo Fisher Scientific, Waltham, MA) was performed according to the manufacturer's instructions. Data are expressed as mean ± SEM (n=3-4 samples/group) and compared by one-way ANOVA and a Tukey's multiple comparison test. ns: not significant, *p < 0.05 versus PBS **(C, D)** Sham mice were treated with OP18 for 20h and blood were collected. **(C)** IL-6 and **(D)** TNFα were measured by ELISA. No significant difference between sham and OP18-treated sham were seen. Data are expressed as mean ± SEM and compared by unpaired two-tailed Student's *t*-test. not significant (ns) vs sham.

Supplemental Figure 7: Effect of OP18 on cytokine production in single DAMP-treated RAW264.7 cells and multiple DAMP-treated THP-1 cells

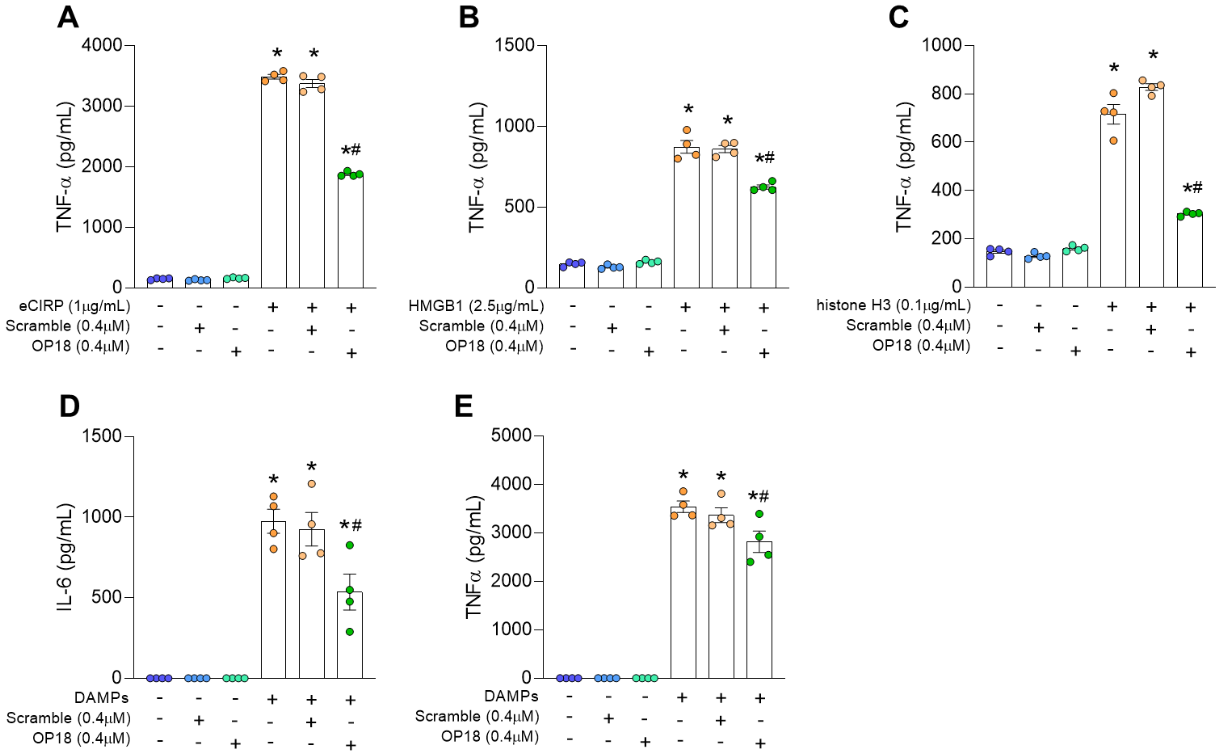

Note: RAW264.7 cells were treated with **(A)** eCIRP, **(B)** HMGB1**,** or **(C)** histone H3 in the presence of OP18 or scrambled peptide. After 20 h, the levels of TNFα in culture supernatants were determined. THP-1 cells were treated with a mixture of DAMPs (1.0 μg/mL eCIRP, 2.5 μg/mL HMGB1 and 0.1 μg/mL histone H3) in the presence of 0.4 μM OP18 or 0.4 μM scrambled peptide. After 20h, the levels of **(D)** IL-6 and **(F)** TNFα in culture supernatants were determined by ELISA kit (IL-6 and TNFα both from BD Biosciences, Franklin Lakes, NJ). Data are expressed as mean ± SEM (n=4 samples/group) and compared by one-way ANOVA and a Tukey's multiple comparison test. *p < 0.05 versus untreated control, #p < 0.05 versus DAMPs alone.
